# Direct Cytosolic Delivery of Citraconylated Proteins

**DOI:** 10.3390/pharmaceutics15010218

**Published:** 2023-01-08

**Authors:** Ritabrita Goswami, Victor Lehot, Yağız Anıl Çiçek, Harini Nagaraj, Taewon Jeon, Terry Nguyen, Stefano Fedeli, Vincent M. Rotello

**Affiliations:** 1 Department of Chemistry, University of Massachusetts Amherst, 710 North Pleasant Street, Amherst, MA 01003, USA; 2 Molecular and Cellular Biology Graduate Program, University of Massachusetts Amherst, 710 North Pleasant Street, Amherst, MA 01003, USA

**Keywords:** cytosolic protein delivery, polymer nanocarrier, citraconic anhydride modification

## Abstract

Current intracellular protein delivery strategies face the challenge of endosomal entrapment and consequent degradation of protein cargo. Methods to efficiently deliver proteins directly to the cytosol have the potential to overcome this hurdle. Here, we report the use of a straightforward approach of protein modification using citraconic anhydride to impart an overall negative charge on the proteins, enabling them to assemble with positively charged nano vectors. This strategy uses anhydride-modified proteins to electrostatically form polymer–protein nanocomposites with a cationic guanidinium-functionalized polymer. These supramolecular self-assemblies demonstrated the efficient cytosolic delivery of modified proteins through a membrane fusion-like mechanism. This approach was validated on five cell lines and seven proteins as cargo. Retention of protein function was confirmed through efficient cell killing via the intracellular enzymatic activity of RNase A. This platform provides a versatile, straightforward, and single-step method of protein modification and efficient direct cytosolic protein delivery.

## 1. Introduction

Cytosolic delivery of protein therapeutics is a promising approach to address several diseases by targeting the many therapeutic targets present in the cytosol [1,2,3,4]. Moreover, access to the cytosol also provides access to other subcellular compartments, such as the nucleus or mitochondria [5]. Access to the cytosol is impeded by the cell membrane which bars entry of negatively charged large exogenous species, such as proteins [6,7,8]. While delivery vehicles can help transport proteins across the cell membrane, most of them employ an endosomal mode of uptake which poses a major challenge [9]. In endocytosis, the protein cargo is sequestered into the acidic vesicular compartment. To perform its activity, the cargo must access the cytosol through endosomal escape, the efficacy of which is still less than 10% [10,11]. Therefore, strategies to deliver proteins directly into the cytosol avoiding endosomal entrapment can pave way for the development of protein therapeutics [12,13,14].

We previously reported direct cytosolic protein delivery using cationic guanidinium-functionalized gold nanoparticles and poly(oxanorbornene) imide (PONI) polymers (PONI Guan) that were self-assembled using proteins expressed with a terminal oligo(glutamate), “E-tags” [15,16]. Electrostatic interactions between the guanidinium groups of the polymer and the carboxylates of the E-tags allowed self-assembly into supramolecular complexes. These nanocomposites performed efficient cytosolic delivery of a set of E-tagged proteins of different sizes and charges, and preserved enzymatic activity. However, both strategies require plasmid engineering to synthesize E-tagged proteins, making the strategy cumbersome. This strategy was adapted very recently to modify native proteins with E-tags through modular biotin–streptavidin bioconjugation [17]. This strategy however requires multiple steps for protein modification and assembly formation.

Protein modification using anhydrides is a straightforward and inexpensive method for covalently modifying target proteins [18,19,20,21]. Here, we demonstrate a facile single-step protein modification method through charge reversal of lysines of native proteins using citraconic anhydride (CA). The nucleophilic amine groups of lysine residues can react with the electrophilic cyclic CA through an addition–elimination mechanism, leading to the ring opening of the cyclic anhydride. Thus, in lieu of protonatable amines, the modified lysine residues bear negatively charged carboxylic acid groups, generating citraconylated proteins with an overall negative charge on the surface of the proteins. These modified proteins were then complexed with PONI Guan to form supramolecular polymer–protein nanocomposites. These nanocomposites enabled efficient cytosolic delivery, as demonstrated through microscopy and nuclear access of citraconylated green fluorescent protein (c−EGFP) in mammalian cells. Efficient cytosolic delivery of six other proteins of varied molecular weights and isoelectic points (pIs), including DsRed, apo-transferrin (apoTf), bovin serum albumin (BSA), ribonuclease A (RNase A), Cre recombinase, and ovalbumin (OVA), confirmed the versatility of the approach. This approach also preserved the enzymatic activity of protein cargos in cells. Citraconylated RNAse A and Cre recombinase (c−RNase A and c−Cre) were delivered in the cytosol leading to efficient cell killing and DNA recombination, respectively. Taken together, this strategy provides a versatile single-step method of protein covalent protein modification and efficient direct cytosolic protein delivery.

## 2. Materials and Methods

### 2.1. Materials

All solvents and chemicals were purchased from Fisher Scientific and Sigma-Aldrich, and used as received, without further purification, unless otherwise stated. All reagents/materials were purchased from Fisher Scientific and used as received. NIH-HEK-293T, HeLa, RAW264.7, NIH-3T3, and MDA-MB-231 (ATCC CRL-3216, CCL-2, TIB-71, CRL-1658, and HTB-26, respectively), were purchased from ATCC. Dulbecco’s Modified Eagle’s Medium (DMEM) (DMEM; ATCC 30-2002) and fetal bovine serum (Fisher Scientific, SH3007103) were used in cell culture.

### 2.2. Methods

**Nanocomposite preparation, characterization, and instrumentation**

A PONI polymer was synthesized following previous reports [16,17] and characterized using ^1^ H Nuclear Magnetic Resonance (NMR) (Figure S1A) and Gel Permeation Chromatography (GPC) (Figure S1B). A solution of the protein of interest (2–10 mg/mL in NaHCO_3_ 0.5 M, pH = 9) was added with a solution of citraconic anhydride in DMSO. The concentration of the anhydride solution was chosen so that the final mixture is about 9:1 (*v/v*) NaHCO_3_ buffer/DMSO. After two hours of incubation and gentle stirring at 25 °C, the remaining small side-products were eliminated by 5 cycles of buffer exchange against Dulbecco’s phosphate buffer saline (DPBS) 1×, pH = 7.4 (using Amicon filters 10 kDa MWCO, Millipore, UFC5010). Isolated yields are typically between 40 and 80%, as determined using a Nanodrop Spectrophotometer. To form nanocomposites, anhydride-modified proteins in DPBS and a guanidinium-functionalized PONI polymer were rapidly mixed (by repeated pipetting, to ensure homogeneous mixing) at the desired Guan/Protein ratio, and incubated at room temperature for ~10 min. For delivery experiments, this mixture was then diluted first with DPBS, and then with the serum-containing culture medium (10% FBS (Gibco), 1% antibiotics (antibiotic–antimycotic, corning), and the final medium/DPBS ratio was 9:1 *v/v*. Size and zeta measurements were performed in 10 mM NaCl and phosphate buffers, respectively, using a Zetasizer Nano-ZS (Malvern). Circular dichroism measurements were performed in a phosphate buffer (without KCl) using a Jasco J-1500. Alamar Blue (Invitrogen) viability studies were performed according to the manufacturer’s instructions, using Molecular Devices Spectramax M2 and SoftMax Pro 7 software.

**Confocal microscopy**

Confocal microscopy imaging experiments were performed using a Nikon TiE stand with an A1 Spectral Detector Confocal and a FLIM/FCS Module. For confocal studies, 2.0 × 10^5^ cells were seeded in a 35 mm imaging dish (Mattek) a day before the imaging experiment. Cell staining was performed 20 min before imaging with Lysotracker Deep Red (Invitrogen) and Hoechst 33,342 solution (Thermofisher, Waltham, MA, USA) to visualize endosomes and nuclei, respectively.

**Imaging flow cytometry**

For flow imaging, we used an Amnis ImageStream MkII Imaging Flow Cytometer (Luminex, Austin, TS, USA). Cells were seeded in a 24-well plate 24 h prior to experimentation at a seeding density of 4.5 × 10^5^ cells/mL and were incubated at 37 °C with 5% CO_2_. The media of the cell was replaced by the nanocomposite samples, prepared as described above on the day of the experiment (500 μL total volume). The cells were incubated at 37 °C with 5% CO_2_ overnight (unless stated otherwise) and then washed with 1× DPBS (twice) and trypsinized. Cells were then fixed using PFA (2%, incubated 20 min at 4 °C), and then placed in a modified flow buffer (Hyclone DPBS + 0.5% BSA, 0.25% NaN_3_, 2 mM EDTA). On the day of the experiment, a 5 mM DRAQ5 fluorescent probe (Thermo Fisher) was added to the samples and allowed to incubate for 20 min. Cells were then processed at a flow rate of 1200 cells/sec and images were taken with 60× magnification. Every imaging flow cytometry experiment was performed in triplicate (three wells per condition), with three experimental replicates collected for each sample at 1000 events per replicate. Error is reported throughout as standard deviation by population. Data were analyzed by Amnis IDEAS software. Focused cell images were isolated through a Gradient RMS histogram of the widefield (Ch01). Single cells were then isolated from debris using a scatterplot of aspect ratio vs. area of the widefield (Ch01). Brightfield images were generated using a dedicated 785 nm laser (SSC).

**Transmission Electron Microscopy (TEM)**

Prepared nanocomposites in small volumes of DPBS were diluted in a phosphate buffer (5 mM) and used directly for TEM imaging. The solution was dip-cast onto a TEM grid (carbon film, 400 mesh copper, and electron microscopy sciences), dried overnight, and samples were imaged using a Tecnai T12 TEM (Thermo-Fisher).

**Protein expression**

Recombinant proteins (EGFP, DsRed, and Cre) were expressed in the E. coli BL21 DE3 PlysS strain (Millipore). Protein expression was carried out in 2xYT media, induced with 1 mM IPTG, and incubated for 16 h at 18 °C. Cells were then harvested and the pellets were lysed using 1% Triton-X-100 (30 min, 37 °C)/DNase-I (New England Biolabs) treatment for 20 min. Digested pellets were centrifuged in polypropylene tubes (Fisher) at 14,000 RPM for 30 min. The supernatant was collected and proteins were purified using HisPur Cobalt columns and dialyzed twice over 18 h in 1× PBS after elution. In the case of Cre recombinase, PBS was supplemented with 300 mM NaCl to limit precipitation. Protein purity was confirmed using SDS-PAGE gel.

## 3. Results and Discussion

### 3.1. Modification of EGFP with CA and Verification of Impact of Modification on Proteins

**Figure 1:**
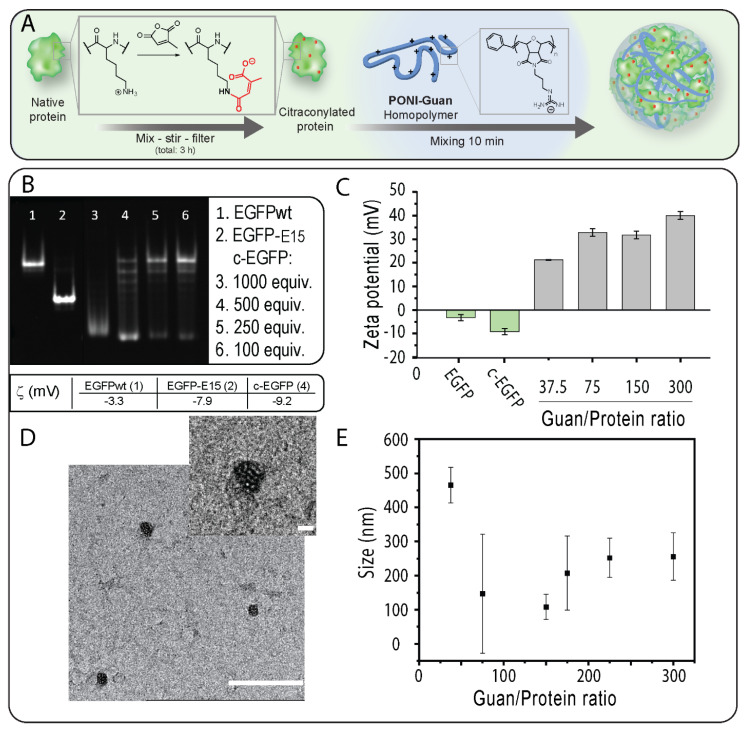
(**A**) c−EGFP can form nanocomposites by self-assembly with a cationic PONI Guan polymer. (**B**) A total of 12% native PAGE analysis of unmodified EGFP, polyglutamate-tagged EGFP (EGFP-E15), and c−EGFP. (**C**) Zeta potential of uncomplexed EGFP and c-EGP, and of nanocomposites at varying Guan/Protein ratios. (**D**) A TEM image of nanocomposites. The scale bar = 500 nm and the inset scale bar = 100 nm. (**E**) Size measurements of nanocomposites at varying Guan/Protein ratios. Data are an average of repeated measurements (n = 3).

We selected EGFP (27 kDa, pI 5.8) as a model cargo because of its fluorescence and its ability to diffuse to the nucleus which provides unambiguous proof of cytosolic localization [5]. We first prepared citraconylated EGFP (c−EGFP) through a reaction with an excess of CA in a bicarbonate buffer (500 mM, pH 9), as previously reported [18] (Figure 1A). Citraconic anhydride was selected for its stability at neutral pH [22]. The addition of negative charges on EGFP upon anhydride modification was observed using native gel electrophoresis (in which the charge of a protein is the main parameter influencing its mobility) [23]. Analysis of EGFP modified using different equivalents (equiv.) of CA showed that 500 equiv. was required to fully convert EGFP to the citrconylated EGFP (c−EGFP) (Figure 1B). Furthermore, the anionic nature of c−EGFP compared with unmodified EGFP was further demonstrated by comparing the zeta potentials, −9.2 and –3.3 mV, respectively (Figure 1B,C). Of note, the zeta potential of genetically engineered EGFP containing a polyglutamate tag, EGFP-E15, used in our previous report [16], was –7.9 mV, indicating that anhydride modification should be sufficient to allow EGFP to form complexes with cationic PONI Guan.

#### Verification Impact of the Modification on Proteins

Covalent modification of proteins can impact their physicochemical properties and biological activities [24]. We then performed circular dichroism on a series of c−EGFP conjugates obtained from different equivalents of CA and compared the results to those obtained from unmodified EGFP (Figure S3). We observed a progressive change in the structure of EGFP when modified with 250 and 500 equivalents of CA, and a complete loss of the native structure when using 1000 equivalents. We thus selected 500 equivalents as the amount of CA to add sufficient negative charges to EGFP without degrading its structure.

### 3.2. Fabrication and Characterization of PONI Guan/c−EGFP Conjugates

After confirming and optimizing c−EGFP modification, incubated c−EGFP samples were then incubated with increasing amounts of the PONI Guan (55 kDa) polymer according to the Guan/Protein ratio. The assemblies were run on native PAGE retardation gel. We found that a minimum Guan/Protein ratio of 38 was necessary to fully complex c−EGFP (Figure S2). The sizes of the assemblies formed at different Guan/Protein ratios were then measured by dynamic light scattering (DLS) (Figure 1E). It was observed that for most formulations, the size of the particles remained between 100 and 300 nm (Figure 1E), with the smallest particles being those formulated at Guan/Protein = 150, which further corroborated with the TEM results (Figure 1D). Importantly, the formed nanocomposites displayed polydispersity index values of 0.6 and 0.8 at Guan/Protein ratios of 37.5 and 75, respectively, and of 0.2 at Guan/Protein ratios of 150 and higher. Hence, the Guan/Protein ratio of 150 was selected for future nanocomposites, as it yielded small-sized, homogeneous complexes.

### 3.3. Cytosolic Protein Delivery, and Nuclear Access of EGFP

**Figure 2:**
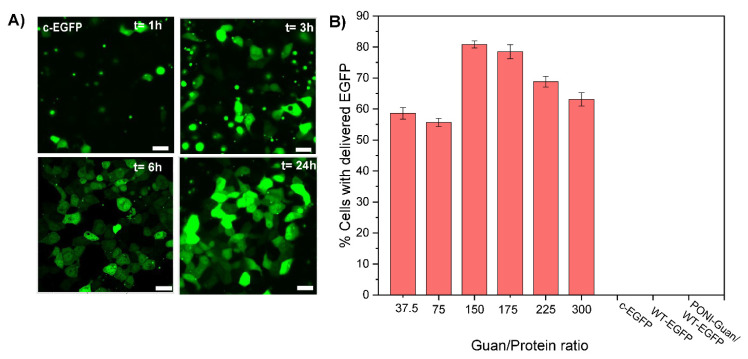
Representative confocal images of HEK-293T and quantification of delivery by flow imaging cytometry after 24 h incubation with PONI Guan/c-EGFP assemblies. (**A**) Delivery of c-EGFP delivery formulated at Guan/Protein ratio 150 (scale bar: 25 µm) at different time points. Diffuse fluorescence and nuclear access, where appropriate, signals cytosolic access. (**B**) Quantification of the percentage of cells delivered with EGFP at different Guan/Protein ratios. Data are an average of replicate measurements (n = 3). Error is the standard deviation by population.

The direct cytosolic delivery of c-EGFP was performed through incubation of PONI Guan/c−EGFP nanocomposites (Guan/Protein ratio = 150) with HEK-293T (human embryonic kidney) cells in culture media (10% FBS) at 37 °C for 1 to 24 h. Confocal microscopy showed diffuse green fluorescence throughout the cytosol (Figure 2A) and the nucleus indicative of cytosolic delivery. Performing the delivery at different time points demonstrated how most delivery events occurred within 3–6 h of incubation (Figure 2A), concurrent with our previous reports [16,17]. Diffuse nuclear fluorescence and co-localization with nuclear stain further with no noticeable overlap with the Lysotracker confirmed the passive diffusion of EGFP in the cytosol and nucleus and direct cytosolic delivery, with no green fluorescence observed from the controls even after 24 h (Figure S4). The assemblies were delivered within ~40 s after attaching to the cell membrane, confirming that the delivery proceeded through a membrane fusion-like process, as found in our previous reports (see supporting video) [15,16]. Furthermore, delivery was only minimally affected by pretreatment with inhibitors of clathrin-mediated endocytosis (chlorpromazine) [25] or macropinocytosis (imipramine [26]) (Figure S5), which supports delivery via a membrane fusion-like mechanism. In contrast, delivery was decreased on pre-treatment with cholesterol sequestration agent methyl-β-cyclodextrin, indicating dependence on cholesterol that is instrumental in membrane fluidity [27]. Delivery was inhibited when incubated at 4 °C for 6 h (Figure S6), which is explained by the contraction and decreased fluidity of the cell membrane at low temperatures [28,29].

We then studied the impact of the formulation of nanocomposites on their delivery. Particles were prepared at various Guan/Protein ratios using c−EGFP and their delivery efficacy was evaluated quantitatively using imaging flow cytometry (Figure 2B). For most formulations, we found that 60–80% of cells were effectively delivered with c−EGFP, with the formulation Guan/Protein = 150, giving the highest delivery efficacy (81% of cells delivered), and nothing was observed for the controls. We thus selected Guan/Protein = 150 as the optimal formulation ratio for c−EGFP.

Taken together, these results show that citraconylated proteins can readily form complexes with PONI polymers, and that these complexes allow for the direct cytosolic delivery of the protein cargo through a membrane fusion-like mechanism. Furthermore, using the delivery of EGFP as a model, we were able to use native PAGE and CD to select the optimal citraconic anhydride equivalents to use in the protein modification step, and then, using DLS and imaging flow cytometry, we could help select the formulation ratio that yields small particles (~108 nm) with a high delivery efficiency (81%). We anticipate that such optimization could be performed for other protein cargos.

### 3.4. Delivery of c−EGFP to Different Cell Lines and Cytosolic Delivery of Different Citraconylated Proteins

**Figure 3:**
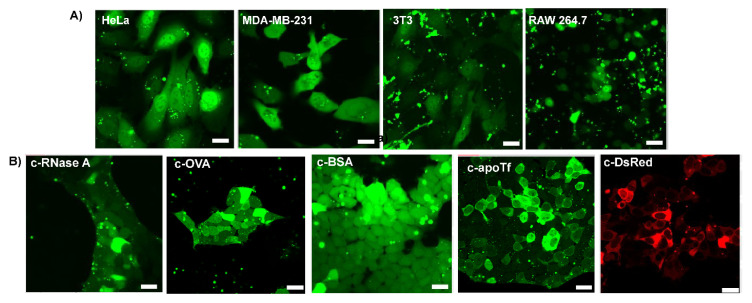
Representative confocal images of delivery with different anhydride-modified proteins after 24 h incubation. The assemblies were delivered at a Guan/Protein ratio of 150 (scale bar: 25 µm). (**A**) Cytosolic delivery of **c−EGFP** to different cell lines. (**B**) Cytosolic delivery of different proteins to HEK-293T cells.

Encouraged by the cytosolic delivery of EGFP in HEK cells, we sought to evaluate the versatility of our platform by performing delivery experiments on cell lines from different tissues and species. We thus delivered c−EGFP to four additional cell lines: HeLa (human cervix adenocarcinoma), MDA-MB-231 (human breast adenocarcinoma), 3T3 (murine fibroblasts), and RAW 264.7 (murine macrophage) cells. In all cases, diffuse cytosolic fluorescence was observed via confocal microscopy (Figure 3A).

To validate the applicability of our delivery system to different proteins, we modified and delivered dye-labeled proteins of different sizes and pI (Table S1), including ribonuclease A (RNaseA, 14 kDa, and pI 8.6), ovalbumin (OVA, 45 kDa, and pI 4.5), bovine serum albumin (BSA, 66 kDa, and pI 4.7), apo transferrin (apoTf, 76 kDa, and pI 6.6), and DsRed (107 kDa, and pI 11.0) to HEK-293T cells. Upon delivery, small proteins, such as RNase and OVA, were diffused through the nucleus, while a dark nucleus was observed when delivering very large proteins (over 100 kDa), such as DsRed (Figure 3B). Interestingly, apoTf (76 kDa), which is only slightly above the commonly admitted nuclear pore complex threshold of 60 kDa, was observed in partial nucleus localization with some cells. This unanticipated result is, however, consistent with previous reports of proteins as big as 90–100 kDa diffusing from the cytosol to the nucleus [30].

### 3.5. Delivery of Functional Enzyme Cre Recombinase A and RNase A

**Figure 4:**
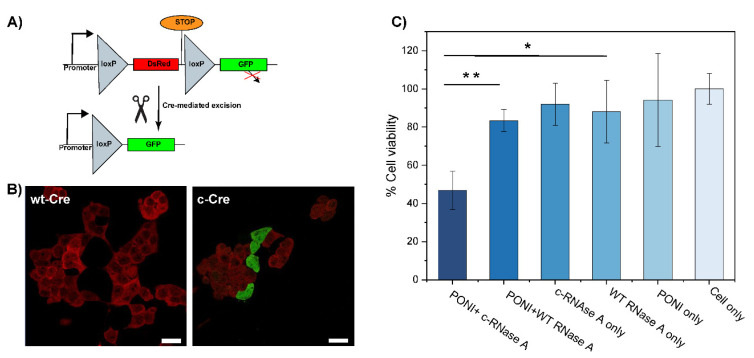
Delivery of citraconylated enzymes. (**A**) Schematic representation of the Cre mechanism of action (**B**) Delivery of c−Cre at Guan/Protein 150 to reporter HEK cell shows ~20% recombination activity (scale bar: 25 µm). Cells displaying red fluorescence are expressing DsRed (no recombination) and cells displaying green fluorescence are expressing GFP (after recombination). (**C**) Delivery of c-RNaseA causes apoptosis. Viability of HeLa cells following PONI Guan/c−RNase A treatment under diverse conditions, as quantified by alamarBlue assay. Data are an average of replicate measurements (n = 3). Error is the standard deviation by population. Statistical analysis was performed through an unpaired t-test; ** *p* < 0.01; * = *p* < 0.05; ns is not significant.

Finally, we applied our co-engineering approach to the delivery of active proteins, Cre recombinase, and RNase A. Cre recombinase is an enzyme that catalyzes the specific recombination between two consensus DNA sequences (LoxP) in the nucleus. It is a popular tool in genome engineering where it is used in a vast variety of organisms [31,32]. To evaluate the activity of c−Cre, we used a Cre reporter HEK-293T cell line which expresses DsRed. A Cre-mediated recombination would excise the DsRed DNA coding sequence and proceed the STOP codon to trigger the expression of GFP, effectively switching the cells from red to green fluorescence (Figure 4A). After 48 h of incubation with c−Cre/PONI nanocomposites (Guan/Protein = 150), we observed that 20% of cells had shifted from expressing DsRed to expressing GFP (Figure 4B). We anticipate that further optimization of the conjugation of Cre would lead to a better compromise between sufficient modification (to allow delivery) and minimal modification (to preserve the enzymatic activity).

RNase A is an endonuclease that can degrade intracellular RNA which, in turn, can trigger apoptosis in cells [33,34]. This pro-apoptotic activity of RNase makes it an interesting anticancer protein cargo. We thus modified RNase A with CA to obtain c−RNase A, which was complexed with PONI (Guan/Protein = 150) to form a nanocomposite. After 48 h of incubation with these nanocomposites (2 µg/mL of RNase A), the viability of HeLa cells was evaluated using the alamarBlue assay. We observed more than 50% apoptosis relative to untreated controls, while less than a 20% decrease in cell viability was observed in groups treated with non-complexed and/or non-modified RNase A (Figure 4C). The slight cytotoxicity observed in control groups, including RNase A, might be due to the non-specific uptake of the protein.

These results demonstrate that functional enzymes could be modified using this approach, complexed, and delivered to the cytosol with retained activity. The discrepancy between the results obtained with c−RNase A and with c−Cre highlights the need for thorough optimization for each protein.

## 4. Conclusions

In summary, anhydride modification of proteins represents an inexpensive and straightforward approach to achieve direct cytosolic delivery using guanidine-functionalized PONI polymers. In less than 3 h, proteins can be modified, purified, and immediately mixed with PONI polymers to form nanocomposites that can then allow their direct cytosolic delivery within a few hours of incubation through a membrane fusion. Cytosolic delivery could be observed by confocal microscopy and quantified by imaging flow cytometry, revealing the high efficiency of our platform. Efficient delivery of c−EGFP to five cell lines (HEK293T, HeLa, MDA-MB-231, 3T3, and RAW264.7), as well as delivery of seven different proteins (EGFP, OVA, apoTf, BSA, RNase A, DsRed, and Cre) to HEK293T, demonstrated the versatility of this platform. The delivery of two functional proteins, Cre recombinase and RNase A, confirmed both the retention of enzymatic activity and cytosolic localization of the delivered proteins. Future work will focus on the more detailed physicochemical characterization of these non-covalent assemblies. In conclusion, anhydride modification using CA represents a facile and rapid method to allow the cytosolic delivery of native proteins.

## Data Availability

Not applicable.

## Supplementary Materials

The following supporting information can be downloaded at https://www.mdpi.com/article/10.3390/pharmaceutics15010218/s1. Figure S1: Characterization of the PONI polymer; Table S1: Molecular weight and isoelectric point (pI) of the delivered proteins; Video S1: Live imaging video for cy-tosolic delivery of c-EGFP by membrane-fusion; Figure S2: Gel retardation assay; Figure S3: Cir-cular dichroism; Figure S4: Representative merged confocal images of c-EGFP delivery to HEK-293T cells after 24 h incubation at 60× magnification; Figure S5: Delivery of c-EGFP under-goes a cholesterol-dependent membrane-fusion type entry pathway; Figure S6: Inhibition of c-EGFP delivery in HEK-293T cells upon incubation for 6 h at 4 °C.
