# Supplementary material for: Direct Cytosolic Delivery of Citraconylated Proteins
Source: Pharmaceutics. 2023 Jan 8;15(1):218. doi: 10.3390/pharmaceutics15010218 (PMC9861219; doi:10.3390/pharmaceutics15010218)
Supplement: Supplementary file 1 [file pharmaceutics-15-00218-s001.zip › pharmaceutics-2102534-supplementary/pharmaceutics-2102534-supplementary.pdf]

Direct Cytosolic Delivery of Citraconylated Proteins

Ritabrita Goswami [1,†], Victor Lehot [1,†], Yağız Anıl Çiçek [1], Harini Nagaraj [1], Taewon Jeon [2], Terry Nguyen [1], Stefano Fedeli [1] and Vincent M. Rotello [1,*]

[1] Department of Chemistry, University of Massachusetts Amherst, 710 North Pleasant Street, Amherst, MA 01003, USA
[2] Molecular and Cellular Biology Graduate Program, University of Massachusetts Amherst, 710 North Pleasant Street, Amherst, MA 01003, USA
* Correspondence: rotello@chem.umass.edu
† These authors contributed equally to this work.

1. Materials
2. Synthesis of polymers
3. Retardation gel for complexation with PONI
4. CD spectroscopy for protein structure
5. PONI-Guan/c-EGFP cytosolic delivery in HEK-293T cells with nuclear staining
6. Live imaging video for c-EGFP delivery by membrane-fusion
7. PONI-Guan/c-EGFP nanocomposite inhibitor studies by flow cytometry
8. Cytosolic delivery with PONI-Guan/c-EGFP at 4 °C
9. Table listing proteins modified with their molecular weights and pIs

1. Materials

All chemicals and solvents for syntheses were purchased from Fisher Scientific and Sigma-Aldrich, and used without further purification, unless otherwise stated. The chemicals were used as received. All reagents were purchased from Fisher Scientific and used as received for chemical synthesis. NIH-HEK-293T, HeLa, MDA-MB-231, RAW 264.7 and 3T3 were purchased from ATCC. Dulbecco's Modified Eagle's Medium (DMEM) (DMEM; ATCC 30-2002) and fetal bovine serum (Fisher Scientific, SH3007103) were used in cell culture, unless otherwise specified. The yields of the compounds reported here refer to the yields of spectroscopically pure compounds after purification.

2. Synthesis of polymer

PONI Guan (55 kDa, PDI=1.02) polymer was synthesized as previously described [1,2] using ring-opening metathesis polymerization and characterized using ^{1}H NMR and Gel Permeation Chromatography (GPC). ^{1}H NMR (400MHz, D_2O) major peaks are 6.05 (br, 2H), 5.86 (br, 2H), 4.94 (br, 2H), 4.56 (br,2H), 3.52 (br, 2H), 3.11 (s, 2H), 1.78 (br, 2H). GPC was performed against Polymethylmethacrylate (PMMA) standards.

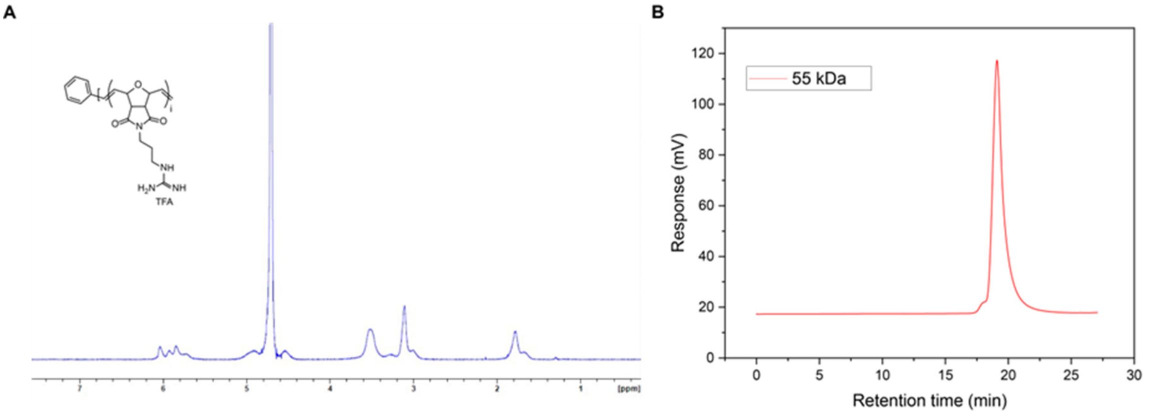

Figure S1: Characterization of the PONI polymer. A) ^{1}H NMR of PONI-Guan in D_2O. B) GPC chromatogram of PONI-Guan 55 kDa polymer against PMMA standard.

3. Retardation gel with PONI

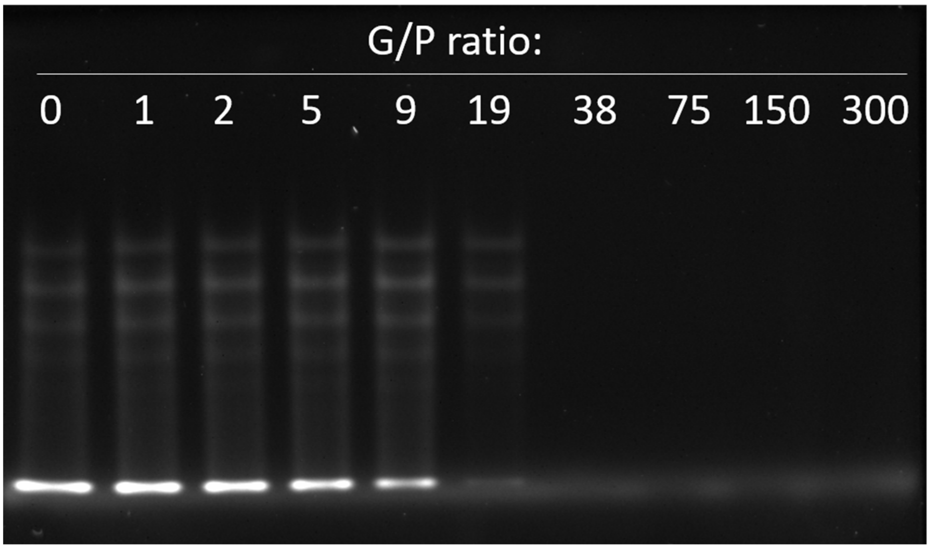

Figure S2: Gel retardation assay: For each well, 2 µg of c-EGFP were mixed with PONI (55kDa) at various G/P ratios and incubated for 10 min before being run on 12% native PAGE.

4. Circular dichroism

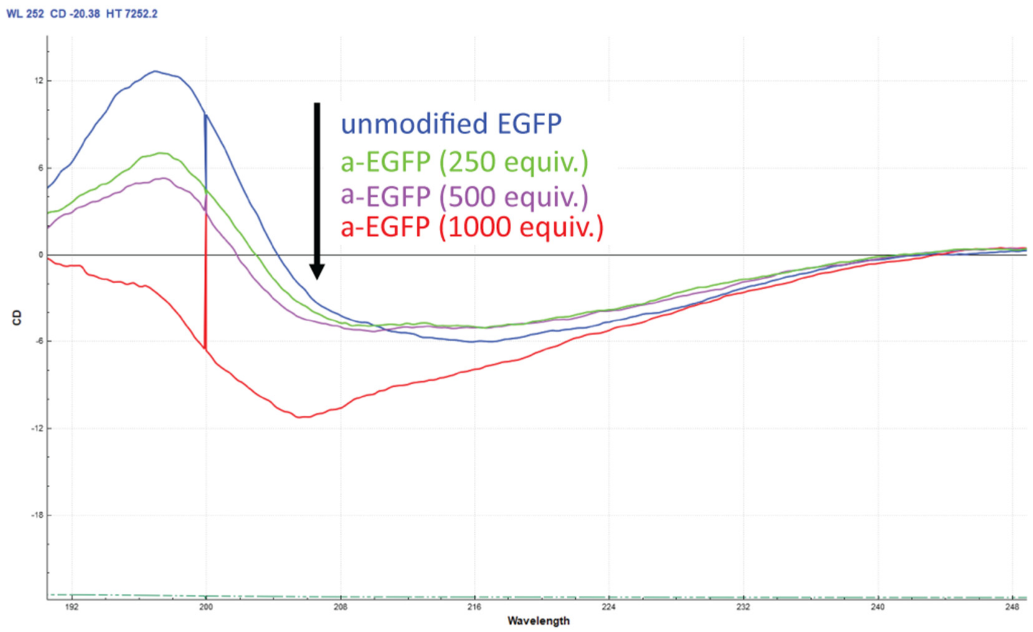

Figure S3: Circular dichroism: Circular dichroism measurements were performed in phosphate buffer (without KCl) using a Jasco J-1500.

5. Cytosolic delivery with nuclear colocalization of PONI-Guan/c-EGFP

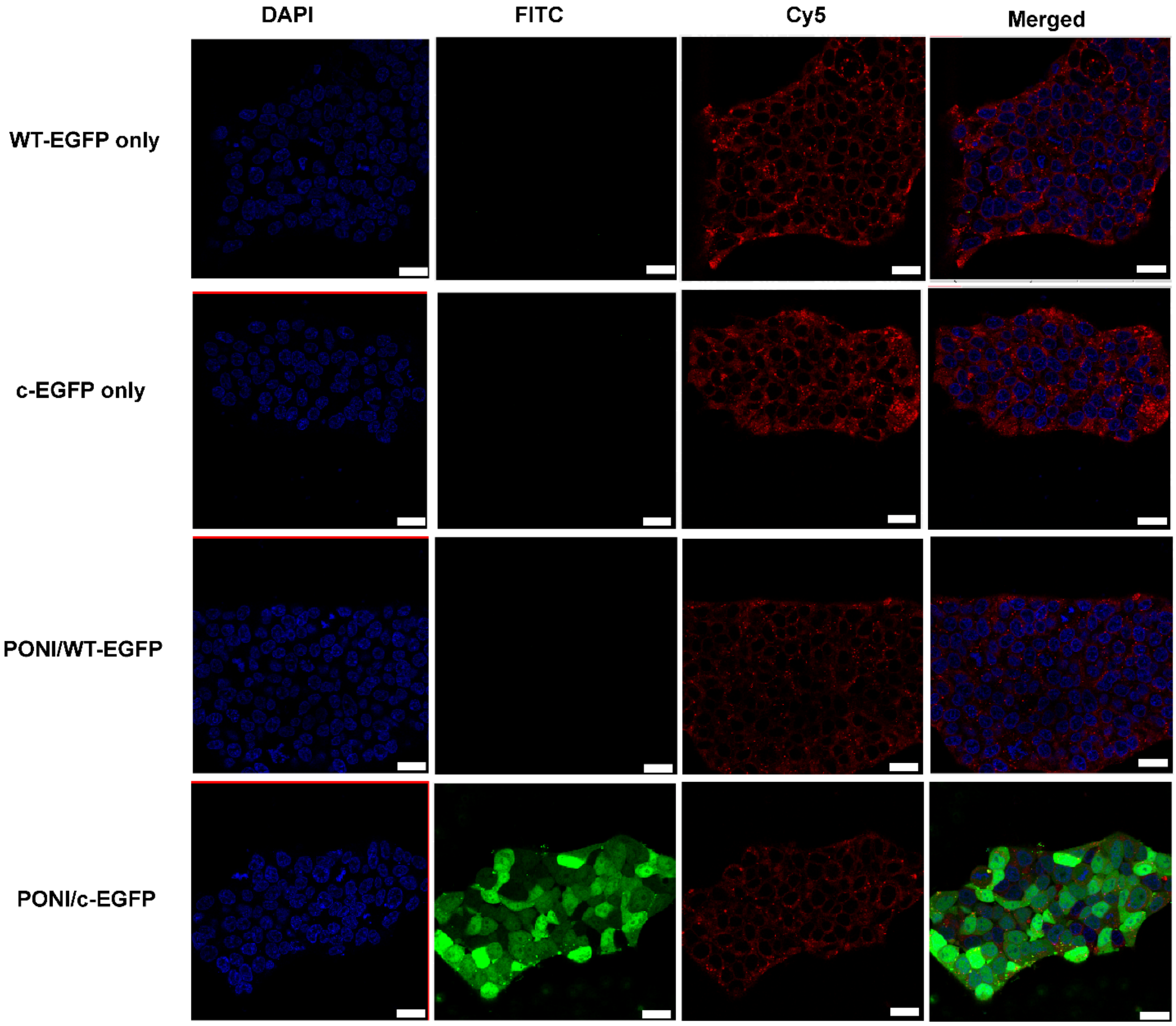

Figure S4: Representative merged confocal images of c-EGFP delivery to HEK-293T cells after 24 h incubation at 60x magnification. Channels displayed are DAPI (Hoechst 33342), FITC (c-EGFP), Cy5 (Lysotracker) and merged channel. Diffuse cytosolic green fluorescence, co-localization with nuclear stain DAPI with no noticeable overlap with Lysotracker indicate cytosolic delivery. Scale bars = 25 µm.

6. Live imaging video for cytosolic delivery of c-EGFP by membrane-fusion

Time-lapse confocal microscopy of HEK-293T cells at 3h time point incubated with PONI-Guan/c-EGFP nanocomposites. EGFP delivers into the cytosol within 40s. Video length: 10 min real-time. Scale bar: 25 µm. See Supporting video.

7. PONI-Guan/c-EGFP delivery in presence of inhibitors

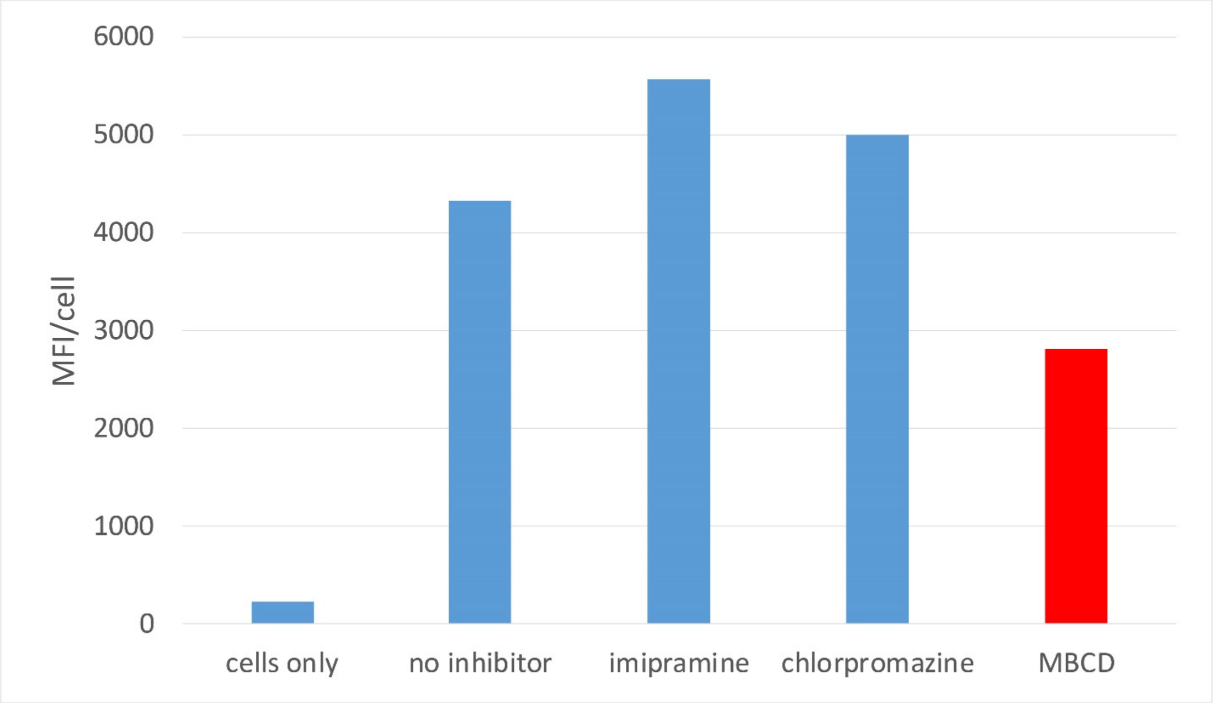

Figure S5: Delivery of c-EGFP undergoes a cholesterol-dependent membrane-fusion type entry pathway. The percentage of cytosolic delivery of EGFP following pretreatment with various small molecules show reduced delivery with methyl-b-cyclodextrin (a cholesterol-depleting agent) while remaining unaffected by other endocytic inhibitors. Data quantified by flow cytometry.

8. PONI-Guan/c-EGFP delivery at 4°C

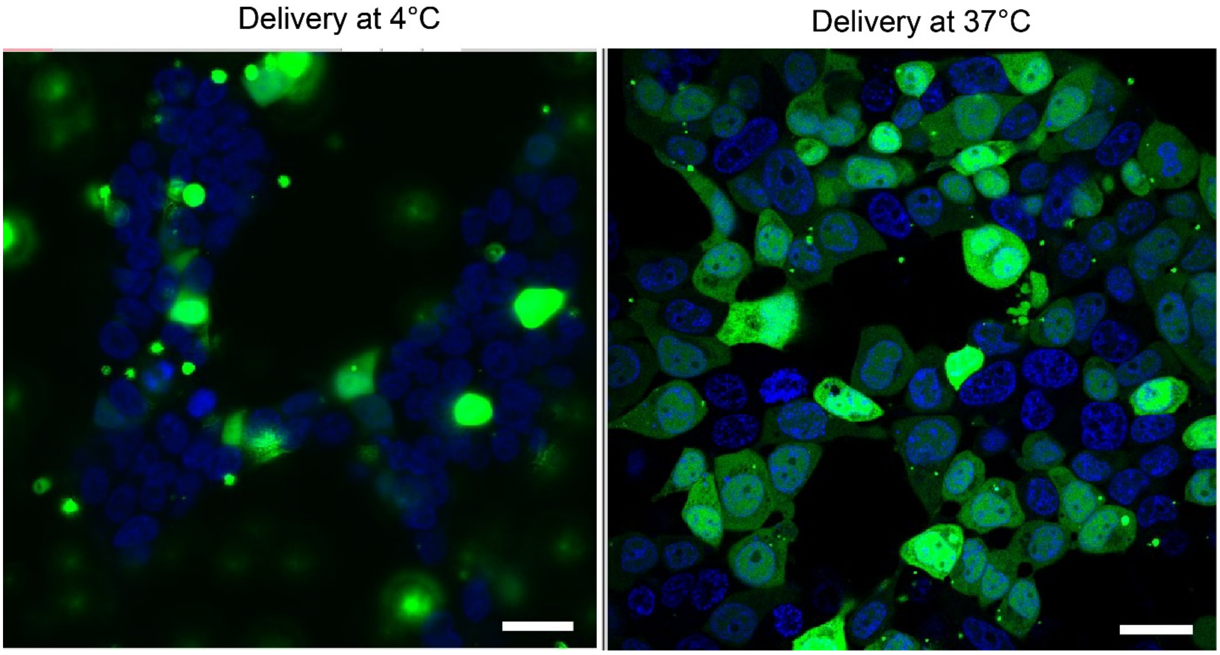

Figure S6: Inhibition of c-EGFP delivery in HEK-293T cells upon incubation for 6h at 4°C. The nuclei are stained with Hoechst 33342 and visible in blue while delivered EGFP is green. Scale bars = 25 μm.

9. Table listing proteins modified with their molecular weights and pIs

Protein	MW (kDa)	pI
EGFP	27	5.8
RNase A	14	8.6
Ovalbumin	45	4.5
BSA	66	4.7
Apo Transferrin	76	6.6
Ds Red	107	11

Table S1: Molecular weight and isoelectric point (pI) of the delivered proteins.

References

1. Lee, Y.W.; Luther, D.C.; Goswami, R.; Jeon, T.; Clark, V.; Elia, J.; Gopalakrishnan, S.; Rotello, V.M. Direct Cytosolic Delivery of Proteins through Coengineering of Proteins and Polymeric Delivery Vehicles. *J. Am. Chem. Soc.* **2020**, *142*, 4349–4355.

2. Luther, D.C.; Lee, Y.W.; Nagaraj, H.; Clark, V.; Jeon, T.; Goswami, R.; Gopalakrishnan, S.; Fedeli, S.; Jerome, W.; Elia, J.L.; et al. Cytosolic Protein Delivery Using Modular Biotin-Streptavidin Assembly of Nanocomposites. *ACS Nano* **2022**,16, 5, 7323–7330.
